# Enhancing Efficiency in Acute Arthroplasty Case Management Through Secure Messaging: A Siilo-Based Multidisciplinary Team (MDT) Audit

**DOI:** 10.7759/cureus.88719

**Published:** 2025-07-25

**Authors:** Ahmed Hamed, Muhammad A Hamid, Islam A Sherif, Mohamed Alsonbaty, Zubair Younis

**Affiliations:** 1 Trauma and Orthopaedics, University Hospitals Birmingham NHS Foundation Trust, Birmingham, GBR; 2 Trauma and Orthopaedics, The Royal Wolverhampton NHS Trust, Wolverhampton, GBR

**Keywords:** complex arthroplasty, multidisciplinary approach (mdt), periprosthetic fractures, periprosthetic joint infection, siilo

## Abstract

Background: Timely decision-making in complex arthroplasty cases such as prosthetic joint infections (PJIs) and periprosthetic fractures is critical to optimizing patient outcomes. Traditional weekly multidisciplinary team (MDT) meetings often delay these decisions. This study evaluates the use of Siilo (Doctolib Siilo, Amsterdam, NLD), a secure medical messaging app, to facilitate expedited MDT discussions in acute arthroplasty admissions.

Methods: A retrospective audit was conducted on acute arthroplasty cases discussed via the Siilo platform over 12 months in 2024. Patient demographics, American Society of Anesthesiologists (ASA) grades, and types of diagnoses were recorded. Time from referral to decision via Siilo was compared to the institutional average wait time for conventional MDT meetings. The primary outcome was time saved in days.

Results: A total of 97 acute arthroplasty admissions were included in the study, with a median age of 72 years. The average time to decision via Siilo was 0.87 days, resulting in a mean time saving of 3.03 days per case. Most patients had ASA grades of 2 or 3, and the most common diagnoses included knee PJI, periprosthetic femoral fractures, and hip PJI.

Conclusion: The Siilo messaging platform reduced time to MDT decision-making in acute arthroplasty cases, demonstrating a practical advantage in managing complex admissions. Secure messaging should be considered as a valuable adjunct to traditional MDT frameworks to enhance clinical efficiency and patient care quality.

## Introduction

In the management of complex arthroplasty cases such as prosthetic joint infections (PJIs) and periprosthetic fractures, timely multidisciplinary team (MDT) input is critical [1,2]. Traditional MDT meetings in the National Health Service (NHS) often occur weekly, introducing delays in clinical decision-making that may negatively impact patient outcomes, length of stay, and hospital resource use [3-5]. With the rise of digital health solutions, secure messaging applications have emerged as promising tools to facilitate quicker, streamlined communication among healthcare professionals [6-9].

Siilo (Doctolib Siilo, Amsterdam, NLD) is a General Data Protection Regulation (GDPR)-compliant secure messaging platform designed for medical professionals to share patient data, images, and case discussions in real time [10]. Its utility in acute care settings via a mobile app that facilitates the creation of ‘cases’ and secure sharing of data and images prompted interest in its role as a virtual MDT tool in our department. This study aims to evaluate the effectiveness of Siilo in accelerating decision-making for acute arthroplasty admissions, instead of waiting for a conventional arthroplasty weekly MDT as occurs normally in our department at the University Hospitals Birmingham NHS Foundation Trust (Birmingham, GBR).

## Materials and methods

University Hospitals Birmingham (UHB) is a large tertiary care center comprising four different hospital sites, with three of them specializing in acute arthroplasty admissions. A weekly complex arthroplasty MDT meeting is conducted every Thursday, where complex acute arthroplasty admissions are also discussed, and multidisciplinary inputs are sought. A retrospective audit was conducted across 12 months (January to December 2024) at our tertiary referral center for complex joint arthroplasty. 

Approval for conducting this clinical audit was duly obtained from the Institutional Clinical Audit and Registries Management Service (CARMS) vide registration no. CARMS-22722. Acute arthroplasty admissions that were discussed via the Siilo platform were included. These included both emergency and urgent elective cases such as PJIs, periprosthetic fractures, dislocations, and implant failures.

Data collected for each case included patient age, sex, American Society of Anesthesiologists (ASA) grade, diagnosis, date of referral, date of decision, and estimated wait time for a standard MDT meeting. This was calculated by noting the day of admission and the number of days from that day to the next Thursday when the conventional MDT would occur. The primary outcome was the time saved in days by using Siilo for MDT discussion. Data was analyzed using Microsoft Excel (Microsoft Corp., Redmond, WA, USA) statistical tools. The time to decision via Siilo was compared to the assumed MDT wait time, and descriptive statistics were used to illustrate trends in ASA grade, case volume, and diagnosis types.

## Results

A total of 97 acute arthroplasty cases were reviewed. The patient cohort had a median age of 72 years (range: 49 to 91). The majority of patients were classified as American Society of Anesthesiologists (ASA) grade 2 (n=37) and ASA grade 3 (n=41). There were 28 patients with periprosthetic fractures, 24 with periprosthetic joint infections, and 29 patients with implant-related complications like dislocation, loosening, or mechanical failure (Tables 1-2). The average time to decision-making using Siilo was 0.87 days, compared to the conventional MDT wait time of 3.9 days, yielding an average time saving of 3.03 days per case.

**Table 1:** ASA grade distribution ASA: American Society of Anaesthesiologists

ASA grade	No. of patients	Percentage
1	4	4.1%
2	37	38.1%
3	41	42.3%
4	15	15.5%

**Table 2:** Breakdown of diagnoses PJI: Periprosthetic joint infection, THA: Total hip arthroplasty, TKA: Total knee arthroplasty

Diagnosis	No. of cases	Percentage
Knee PJI	16	16.5%
Periprosthetic fracture (post-THR/TKR)	28	28.9%
Hip PJI	8	8.2%
Other infections	14	14.4%
Dislocation	12	12.4%
Implant loosening	9	9.3%
Mechanical failure	8	8.2%
Other	2	2.1%

## Discussion

This audit highlights the considerable impact of secure mobile messaging on improving efficiency in complex orthopaedic care. The primary finding, a mean reduction of 3.03 days in decision-making time, demonstrates how mobile messaging tools can bridge the communication gap that exists between weekly MDTs. This is especially significant in cases involving ASA grade 2 and grade 3 patients, who comprised nearly 82% of the cohort and are most vulnerable to adverse outcomes from delays [4].

The use of secure messaging has the potential to fundamentally change inpatient management and improve our ability to care for complex hospitalized patients by facilitating efficient communication [11]. Moreover, the quality of communication via a secure messaging system between healthcare professionals highlights its versatility in tackling a range of issues. In our app, the ability to create ‘cases’ for discussion and the ability to return to a complex case in the future to share progress, follow-up, etc. makes communication and information sharing seamless, quick, and easy to access. In addition to this, rapid decision-making also allows for better resource allocation, reduces inpatient bed-days, and facilitates earlier interventions.

These findings align with broader literature supporting digital communication in clinical decision-making. In their 2019 study, Liu et al. conducted a comprehensive evaluation of secure messaging applications within a healthcare system, aiming to identify platforms that not only ensure Health Insurance Portability and Accountability Act (HIPAA) compliance but also enhance clinical collaboration and workflow efficiency. The researchers assessed 19 vendors, categorizing them into three tiers based on functionalities: basic secure communication, integration within existing clinical applications, and dedicated communication and collaboration systems. Essential features identified included message encryption, user authentication, and integration capabilities with electronic health records. The study emphasized the importance of considering factors such as mobile device management, bring-your-own-device policies, and Wi-Fi infrastructure when implementing secure messaging solutions. The authors concluded that secure messaging applications, when properly evaluated and implemented, can significantly improve communication efficiency and clinical workflows in healthcare settings [12]. A case study evaluating secure messaging applications found improvements in communication efficiency and collaboration within a ‘virtual’ heart team. Their report was on their experience with instant messaging to establish a virtual heart team between cardiologists and cardiac surgeons at remote hospitals [13].

In their prospective, randomized controlled study on the comparison of secure messaging versus standard telephone usage for consultations in the emergency department (ED), Gulacti et al. found that the use of secure messaging for communication significantly reduced ED length of stay as well as consultation time, greatly helping improve patient flow in the ED [14]. Knees et al., in their study on secure messaging platforms for inpatient communication, noted that these platforms helped streamline workflows, enabled better coordination across MDTs, and improved patient safety. However, they also remarked on its potential to disrupt workflows through overwhelming message volumes, task interruptions, and considerable risk of miscommunication [11].

While this study is limited by its retrospective design, the magnitude of time saved presents a compelling argument for digital MDT integration. Evaluating user experience and identifying barriers to adoption across disciplines will also be crucial for scaling this model across other specialties.

## Conclusions

The use of a secure mobile messaging service for multidisciplinary decision-making in acute arthroplasty care greatly reduced time-to-decision by over three days per case. With consistent use across a range of complex arthroplasty presentations, the use of secure app-based messaging demonstrated its potential to enhance efficiency in patient care. Secure messaging platforms should be considered a viable adjunct to traditional MDT frameworks in high-acuity clinical environments.
